# Differential Diagnosis of Malaria on Truelab Uno^®^, a Portable, Real-Time, MicroPCR Device for Point-Of-Care Applications

**DOI:** 10.1371/journal.pone.0146961

**Published:** 2016-01-19

**Authors:** Chandrasekhar Bhaskaran Nair, Jagannath Manjula, Pradeep Annamalai Subramani, Prakash B. Nagendrappa, Mulakkapurath Narayanan Manoj, Sukriti Malpani, Phani Kumar Pullela, Pillarisetti Venkata Subbarao, Siva Ramamoorthy, Susanta K. Ghosh

**Affiliations:** 1 bigtec Private Limited, 2nd Floor, Golden Heights, 59th C Cross, 4th M Block, Rajajinagar, Bangalore, 560 010, Karnataka, India; 2 National Institute of Malaria Research, ICMR Complex, Poojanahalli, Kannamangala Post, Devanahalli, Bangalore, 562 110, Karnataka, India; 3 Institute of Trans-disciplinary Health Sciences and Technology (IHST), FRLHT, 74/2, Jarakabande Kaval, Attur post, Via Yelahanka, Bangalore, 560 106, Karnataka, India; 4 School of Bio Sciences and Technology, School of Advanced Sciences VIT University, Vellore, Tamil Nadu, 632014, India; Centro de Pesquisa Rene Rachou/Fundação Oswaldo Cruz (Fiocruz-Minas), BRAZIL

## Abstract

**Background:**

Sensitive and specific detection of malarial parasites is crucial in controlling the significant malaria burden in the developing world. Also important is being able to identify life threatening *Plasmodium falciparum* malaria quickly and accurately to reduce malaria related mortality. Existing methods such as microscopy and rapid diagnostic tests (RDTs) have major shortcomings. Here, we describe a new real-time PCR-based diagnostic test device at point-of-care service for resource-limited settings.

**Methods:**

Truenat^®^ Malaria, a chip-based microPCR test, was developed by bigtec Labs, Bangalore, India, for differential identification of *Plasmodium falciparum* and *Plasmodium vivax* parasites. The Truenat Malaria tests runs on bigtec’s Truelab Uno^®^ microPCR device, a handheld, battery operated, and easy-to-use real-time microPCR device. The performance of Truenat^®^ Malaria was evaluated versus the WHO nested PCR protocol. The Truenat^®^ Malaria was further evaluated in a triple-blinded study design using a sample panel of 281 specimens created from the clinical samples characterized by expert microscopy and a rapid diagnostic test kit by the National Institute of Malaria Research (NIMR). A comparative evaluation was done on the Truelab Uno^®^ and a commercial real-time PCR system.

**Results:**

The limit of detection of the Truenat Malaria assay was found to be <5 parasites/μl for both *P*. *falciparum* and *P*. *vivax*. The Truenat^®^ Malaria test was found to have sensitivity and specificity of 100% each, compared to the WHO nested PCR protocol based on the evaluation of 100 samples. The sensitivity using expert microscopy as the reference standard was determined to be around 99.3% (95% CI: 95.5–99.9) at the species level. Mixed infections were identified more accurately by Truenat Malaria (32 samples identified as mixed) versus expert microscopy and RDTs which detected 4 and 5 mixed samples, respectively.

**Conclusion:**

The Truenat^®^ Malaria microPCR test is a valuable diagnostic tool and implementation should be considered not only for malaria diagnosis but also for active surveillance and epidemiological intervention.

## Introduction

Malaria is a parasitic disease that is endemic in most tropical and sub-tropical ecosystems worldwide with half of the world’s population at risk of contracting malaria [1, 2]. As per the most recent World Health Organization (WHO) estimates released in December 2014, there were about 198 million cases of malaria and an estimated 584,000 malaria deaths in 2013 [2]. Despite control measures, malaria related morbidity and mortality remain significantly high in many developing countries.

Malarial parasites belong to the genus *Plasmodium* and infect many vertebrate hosts, including several species of non-human primates. Five *Plasmodium* species are parasitic to humans: *P*. *falciparum*, *P*. *malariae*, *P*. *ovale*, *P*. *vivax* and *P*. *knowlesi*. Of these, *P*. *vivax* and *P*. *falciparum* are associated with most malaria morbidity and mortality. *P*. *falciparum* is the most dangerous and can cause medically severe form [3]. For proper malarial treatment, accurate and rapid diagnosis is essential. It is also essential to have sensitive and specific malaria diagnostic tools to prevent over-treatment and injudicious use of anti-malarials [4].

Malaria diagnosis is done using tools like microscopy, rapid diagnostic tests (RDTs) involving parasite antigen/enzyme detection and molecular tools (nucleic acid-based). The microscopic examination of a blood smear is considered as “gold standard” for malaria diagnosis in endemic countries. However, the method is laborious and inconsistent [5], with high inter-observer variability [6]. Moreover, microscopic examination demands significant infrastructure to ensure quality, which is not always possible. Microscopy also has limited sensitivity which means that many samples with low parasitic loads will be incorrectly diagnosed as false negatives. RDTs are widely used for malaria detection due to their ease of use. However, the RDT method does not offer improved sensitivity over microscopy, in fact, the sensitivity decreases where parasitaemia level is below 100 per μl [7].

Polymerase Chain Reaction (PCR) is a popular nucleic acid based tool that is used for infectious disease diagnosis. PCR is known to be highly sensitive and specific. In various studies, it has consistently revealed the widespread presence of infections with parasite densities below the threshold level of detection of either microscopy or RDTs [8,9,10].

The barriers to adoption of PCR include its high cost, and the amount of infrastructure required in terms of equipment and a sophisticated laboratory setup with stable power and refrigerators for reagent storage. Most modern commercial PCR machines are not portable or battery-operated, and so are unsuitable for field or point-of-care applications, especially in areas with low resources. PCR is thus sparingly used in areas of high malaria burden which are largely found in the developing world. Efforts have been made to simplify nucleic acid-based testing by using, for example, isothermal amplification technologies such as LAMP or simple and cheap read-out systems, such as nucleic acid lateral flow immuno-assay (NALFIA) [11]. The NALFIA requires an additional amplification step and is intended to be coupled with an isothermal amplification method like LAMP. A major drawback of LAMP is that it is prone to contamination and amplification of non-targeted DNA sequences, which lowers the specificity of the assay. There is an urgent need for a nucleic acid-based malaria test that is highly sensitive and specific, and can be used at point-of-care in resource-limited settings.

Here, in the current study we describe the Truenat^®^ Malaria test (bigtec Labs, Bangalore, India) a novel, simple and cost-effective point-of-care technique for differential *P*. *falciparum* (PF) and *P*. *vivax* (PV) malaria diagnosis using the Truelab Uno^®^ device (bigtec Labs, Bangalore, India). The Truelab Uno^®^ device is a portable, light weight, real-time PCR based nucleic acid detection device, operated using a re-chargeable battery power source.

**Fig 1:**
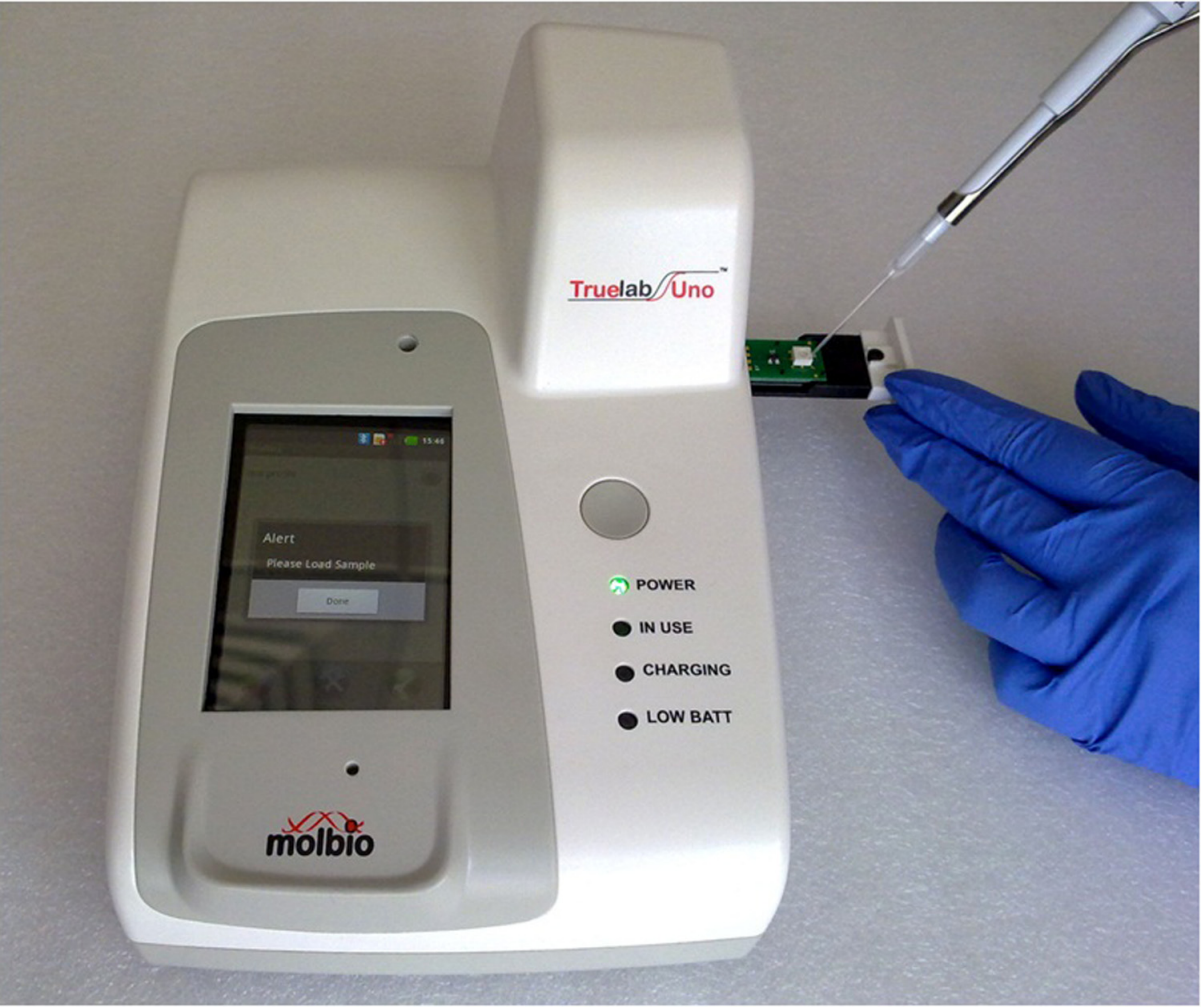
Addition of 5μl of DNA to Truenat Malaria chip on Truelab Uno^™^ microP.

The device weighs 900 grams, and its dimensions are 210 mm X 140 mm X 109 mm (length x breadth x height). It consists of a portable unit housing, an optical detection system, the electronic components controlling different aspects of the unit [12], a 3.2 inch touch-sensitive screen and a sample holder for the PCR chip (Fig 1). The specific PCR program required to be run is selected using the touch screen. The software allows real-time monitoring of thermal cycling and PCR amplification. It uses a disposable microchip [13], with pre-loaded, room temperature stabilized PCR reagents—enabling the user to just add the purified nucleic acid sample and start the test. The chip has flash memory which stores test related information including the batch-specific standard curve written in the form y = mx + c, where m represents the gradient and c represents the y intercept. At the end of a test, the results are displayed on the touch screen, with the device automatically calculating the pathogen load by plugging in the Ct (x) obtained into the standard curve equation. The device is GPRS/Wi-Fi/Bluetooth enabled, to aid in result transfer and automated disease surveillance efforts. The device can also store up to 5000 test results which users can access at a later date.

The use of the Truelab Uno^®^ platform and Truenat^®^ microchips has been reported previously in the detection of Tuberculosis in near-care settings in India [14, 15]. These studies compared the use of the Truelab Uno^®^ platform against reference standards used in the field, and found that the platform was suited to use in point-of-care settings in terms of accuracy, ease-of-use, turn-around time and robustness of the device.

In this study, the development of a novel PCR-based malaria test that runs on the Truelab Uno^®^ platform is described. The performance of the Truenat Malaria test on the Truelab Uno platform compared to expert microscopy and a RDT is also reported.

## Materials and Methods

### Truenat^®^ Malaria

The proprietary Truenat Malaria assay was developed and standardized by bigtec. The target genes for both PF and PV are single copy genes. The assay is based on Taqman chemistry. The probes and primers were designed and characterized using tools like Primer Express (Applied Biosystems), Primer3 (simgene.com/Primer3) and NCBI Primer Blast (www.ncbi.nlm.nih.gov/tools/primer-blast/) ClustalW software (http://www.clustal.org/clustal2/) was used for *in silico* sequence alignment analysis. The assay was first standardized and characterized on a conventional real-time PCR device (ABI 7500, Applied Biosystems) and then ported to the Truelab Uno^®^ system where the PCR reaction happens on a microchip. The final result after the Truenat^®^ Malaria test is run on the Truelab Uno^®^ system is provided as genome equivalents per μl for positive samples.

### Performance evaluation of Truenat^®^ Malaria assay versus the WHO nested PCR protocol

A panel of 100 archived samples was used to test the sensitivity and specificity of the Truenat Malaria test against the WHO nested PCR protocol [4]. The panel contained 50 PF positive samples and 50 PV samples as classified by the WHO nested PCR protocol (performed on ABI 7500, Applied Biosystems). These 100 archived samples were used as a pilot panel to assess the performance of the Truenat Malaria assay versus a molecular reference standard. 100μl of each blood sample was processed using the QIAamp DNA blood mini kit (Qiagen). The extracted DNA was subjected to nested PCR and on a conventional thermal cycler (ABI 7500) Applied Biosystems followed by gel electrophoresis according to SOP: 5.8 of WHO; Methods Manual for Product Testing of Malaria Rapid Diagnostic Tests, Ver 2, 2009. The result of the WHO nested PCR protocol is qualitative—samples are detected as positive or negative only, no information is obtained on the parasitaemia. The same elute was also tested on the ABI 7500 using the Truenat^®^ Malaria mastermix and on the Truenat^®^ Malaria chips as described in the respective section.

### Study design

This cross-sectional paired study design was chosen to determine the performance of the Truelab Uno^®^ microPCR in patients with symptoms of malaria in comparison with smear expert microscopy and a national program approved Rapid Diagnostic Test kit (*FalciVax*^®^, Tulip Group, Goa, India) [4]. A team from National Institute of Malaria Research (NIMR) collected patient samples and prepared blood smears for expert microscopy, a second team from NIMR performed the RDT-based diagnosis and the team from bigtec performed the PCR analysis. The tests were performed independently by each team; results of other tests were kept hidden until all samples were analyzed by all three methods. Before conducting the trial, a training programme was conducted for all the staff involved in the study. Good clinical and laboratory practices were followed.

### Study population and specimens

A total of 281 patients were recruited for this study. Study samples were collected from Wenlock Government District Hospital, Mangalore, Karnataka from August 2009 to October 2010. Samples were also collected from two malaria endemic villages in Koraput district, Odisha from July to November 2009. Patients of either sex, from 3 to 62 years of age, presenting with fever or recent history of fever, were chosen for participation in the study. Finger pricked whole blood samples (approximately 50μl) were collected in EDTA coated vials and stored at -20°C during this period. The DNA from blood samples was extracted using QIAamp DNA blood Mini kit.

### Expert microscopy

Finger pricked thick and thin whole blood smears were prepared on the same slide. The thin smears were fixed with Methanol and the thick smears were dehaemoglobinized with distilled water. These smears were stained with 10% Giemsa. Subsequently, these stained smears were examined on double blind mode independently by two expert microscopists. Any discordant result was resolved by a Senior Scientist (SKG). Final readings were calculated on the basis of average of the two microscopists. A smear was declared negative after thorough scanning of 100 micro-fields on thick films. Quality control was performed by randomly choosing 10% of the smears for analysis by the Senior Scientist (SKG). Parasitaemia was calculated against 200 white blood cells on thick film assuming 8000 WBCs/μl blood for a single patient [16].

### RDT protocol

*Falcivax*^™^ is a rapid, qualitative, two site sandwich immunoassay utilizing whole blood for the detection of *P*. *falciparum* specific histidine rich protein-2 (*Pf*HRP-2) and *P*. *vivax* specific pLDH. The test can also be used for specific detection and differentiation of *P*. *vivax* and *P*. *falciparum* malaria [17]. Test was performed as per the instruction provided with the kit. In brief, 5 μl blood was added on to the application pad following adding of six drops of buffer solution. Final reading was taken after 20 minutes. Positive for respective malaria parasite or negative results were obtained based on color code mentioned in the strip. This test was performed by an expert technician.

### Sample collection and DNA isolation

Blood samples were collected from suspected malaria patients residing in endemic areas in EDTA vials using finger prick method. After completing the microscopic examination and RDT, the remaining blood was used for PCR analysis. DNA was isolated from the remaining blood samples using QIAamp DNA blood mini kit according to the instructions as per the handbook of QIAamp DNA blood Mini kit. For amplicon purification from agarose gels, QIAquick gel extraction kit was used as per manufacturer’s instructions. Proprietary malaria-specific primers and probes were synthesized by Sigma. The master-mixes used for PCR are proprietary components of the Truenat Malaria kit.

### Real-time PCR on commercial ABI 7500 machine & Truelab Uno^™^ microPCR

Real-time PCR on ABI 7500: 4 μl of extracted DNA was mixed with 6 μl of the Truenat malaria mastermix and real-time PCR was performed on ABI 7500 (Applied Biosystems, USA) under the following cycling conditions:

Step 1: 95°C for 60 sec
Step 2: 95°C for 5 sec
Step 3: 60°C for 34 sec

Steps 2 & 3 were repeated for 40 cycles

Real-time PCR on Truelab Uno^™^ microPCR: 5μl of extracted DNA was added to the Truenat malaria microchip and the real-time PCR was done using a pre-programmed profile on the Truelab Uno^™^ microPCR. Results were observed on the screen and compared to the results obtained on the ABI 7500 using the same mastermix.

### Relative standard curves for *P*. *falciparum* and *P*. *vivax* quantification

Genomic DNA from *P*. *falciparum* and *P*. *vivax* clinical samples was used as starting material to generate the relative standard curves for qPCR. 100 μl of whole blood was processed using the QIAamp DNA blood mini kit. 25 μl of the extracted DNA was subjected to PCR along with the respective primers using a conventional PCR machine (ABI 7500, Applied Biosystems). After PCR the amplified samples were run on an agarose gel and stained with ethidium bromide. The amplicon band was then excised from the gel and purified using a Qiaquick gel extraction kit. The absorbance (2μl of DNA) was estimated at 260nm using a NanoDrop 2000 (Thermo Fisher Scientific Inc, USA) following the manufacturer’s instructions. The copy number of the pure amplicon was calculated as given below. Serial dilutions of a known amount of amplicon DNA were used to produce a standard curve in the form y = mx + c, where m represents the gradient and c represents the y intercept. The PCR was performed in triplicates on the Truenat Malaria chips run on the Truelab Uno system and the average Ct was used for plotting the standard curve. The standard curves for the PF and PV were flashed onto the Truenat^®^ Malaria chips in order to convert the Ct obtained to genome equivalents/μl.

Nanomoles of amplicon were calculated using the following equation:

nmoles/ml = 1000 × OD260 (1cm) × 1 ml (vol) / Extinction coefficient of amplicon

Extinction coefficient of the DNA was calculated from individual base coefficient by summing up.

Copy number was calculated using the formula:

Copy number /ml = (Moles/ml) × Avogadro number

### Lower limits of detection

The WHO international standard for *P*. *falciparum* DNA nucleic acid amplification technology (NAT) assays, obtained from the National Institute for Biological Standards and Control (NIBSC; Hertfordshire, United Kingdom) was used as the calibration reference reagent for the *P*. *falciparum* assay. The standard consists of a freeze-dried whole blood preparation collected from a patient by exchange transfusion. As per instructions in the product data sheet, this lyophilized material was suspended in 500 μl of sterile, nuclease-free water to a final concentration of 10^9^ IU/ml. As per a previous study [18], this corresponds to a parasitaemia of 9.79 parasites/100 red blood cells and an estimated parasite density of 469,920 parasites/ml (after reconstitution), based on average red blood cell count. Serial dilutions of the stock were subjected to PCR in replicates in order to arrive at the lowest concentration of the *P*. *falciparum* reference material detectable by the Truenat^®^ Malaria assay.

For estimating the lower limit of detection of *P*. *vivax*, in absence of a reference material, genomic DNA was used. The extraction and quantitation of the genomic DNA are described in Section 2.9. Serial dilutions of the DNA were subjected to PCR in replicates to estimate the lowest number of *P*. *vivax* genome equivalents that were detectable by the Truenat^®^ Malaria assay.

### Comparison of the performance of Truelab Uno^™^ microPCR with that of expert microscopy and RDT

In order to determine the specificity and sensitivity of Truelab Uno^™^ microPCR with expert microscopy and rapid diagnostic test (RDT), which are the standard tests for malaria detection, a study was done on a sample panel consisting of 281 clinical samples including 6 follow-up samples (samples from patients who already had malaria and are undergoing treatment) collected from two different malaria endemic areas. The samples were subjected to malaria detection by expert microscopy, RDT and Truenat Malaria test on Truelab Uno^™^ microPCR by different technicians who did not know the identity of the samples prior to testing. The data was compiled by NIMR and the results were unblinded by an expert committee. The percentage sensitivity of the Truenat^®^ Malaria and the RDT tests using microscopic examination of the blood smears as the gold standard was calculated as follows: true positives/(true positives + false negatives) × 100. In addition, 95% Confidence Intervals (95% CI) were calculated using an online calculator http://vassarstats.net/clin1.html.

### Ethics

The study was approved by Institutional Ethics Committee of Kasturba Medical College, Mangalore—a constituent college of Manipal University, Karnataka, India. The institutional Review Board approved the protocol, which was implemented in accordance with the Helsinki Declaration and regulatory guidelines of ICMR, Government of India. The study protocol was also approved by the Scientific Advisory Committee of National Institute of Malaria Research (ICMR), New Delhi. Finger pricked blood samples were collected from all participants after obtaining written informed consent by themselves or their parents or guardians. Written assent was also taken from the guardians of children who were able to understand the study process, when required. Truelab Uno results were not used for clinical decision making and the patient samples used were anonymous with no identifiers to patient identity.

## Results

### Linear range

Based on the standard curves generated using genomic DNA, the Truenat Malaria assay was found to be linear over 6 orders of magnitude for *P*. *falciparum* and *P*. *vivax* detection and quantification

### Lower limits of detection

Based on the use of the WHO international standard for *P*. *falciparum* DNA nucleic acid amplification technology (NAT) assays, obtained from NIBSC, the lower limit of detection for *P*. *falciparum* is estimated to be approximately 4.7 parasites/μl. With the use of the DNA from a *P*. *vivax* positive clinical sample, the lower limit of detection of the *P*. *vivax* assay was found to be 10 genome equivalents per PCR reaction. Based on the relation between genome copy number and parasitic load (parasites/μl) established in a previous study [19], the limit of detection of Truenat^®^ Malaria is estimated to 1.3 parasites/μl for *P*. *vivax*.

### Sensitivity and Specificity of Truenat Malaria using a Nucleic Acid based reference standard

The nested PCR protocol provides qualitative (positive/negative results only). So for comparison, Truenat^®^ results were categorized as positive or negative. The parasitaemia information obtained (from coversion of Ct into parasites/μl based on the standard curve) was ignored for this analysis. The Truenat Malaria test identified all 50 *P*. *falciparum* positive samples correctly and was negative for *P*. *vivax* in all of these samples. Similarly, the Truenat Malaria test identified all 50 *P*. *vivax* positive samples and did not detect any of these as *P*. *falciparum* positive. This gave an analytical sensitivity and specificity of 100% each at the species level (Tables 1 and 2).

**Table 1:** Results of Truenat Malaria tests versus the WHO nested PCR protocol for *Plasmodium falciparum* detection

Device	*P*. *falciparum*	WHO nested PCR protocol	
		*P*. *falciparum* positive	*P*. *falciparum* negative
**Truenat**^**™**^ **Malaria**	***P*. *falciparum* Positive**	**50**	**0**
	***P*. *falciparum* Negative**	**0**	**50**

**Table 2:** Results of Truenat Malaria tests versus the WHO nested PCR protocol for *Plasmodium vivax* detection.

Device	*P*. *vivax*	WHO nested PCR protocol	
		*P*. *vivax* Positive	*P*. *vivax* Negative
**Truenat**^**™**^ **Malaria**	***P*. *vivax* Positive**	**50**	**0**
	***P*. *vivax* Negative**	**0**	**50**

### Real-time PCR on commercial ABI 7500 machine & Truelab Uno^®^ microPCR

The PCR was performed in parallel on the ABI 7500 and the Truelab Uno^®^ to verify whether the result obtained on the Truelab^®^ was the same as that obtained using a commercial thermal cycling unit. It was found that the results obtained from the Truelab Uno^®^ microPCR were the same as that obtained using the commercial ABI 7500 machine for all 281 samples, with no device related artifacts introduced by the thermal cycling process on the Truelab Uno^®^. The overall time taken to complete the PCR starting from DNA isolation was approximately about 45 mins with the Truelab Uno^™^ microPCR device while it was around 1hr and 30 mins with the commercial ABI 7500 machine.

### Comparison of the performance of Truelab Uno^™^ microPCR with that of expert microscopy and RDT

Of the 281 samples, 141 were negative for both *P*. *falciparum* and *P*. *vivax* by expert microscopy. Of these 141 negative samples, 117 were negative for both *P*. *falciparum* and *P*. *vivax* by Truenat Malaria, and 129 were negative for *P*. *falciparum* and *P*. *vivax* by RDT (S1 Table).

Expert microscopy detected 140 samples as either *P*. *falciparum* positive (65 of 140) or *P*. *vivax* positive (71 of 140), or mixed infection with both *P*. *falciparum* and *P*. *vivax* positive (4 of 140). The Truenat Malaria test identified 64 of 65 *P*. *falciparum* positive samples, all 71 *P*. *vivax* positives and all 4 mixed infection samples correctly giving a sensitivity of 99.3% at the species level (Table 3). The RDT detected 63 of 65 *P*. *falciparum* positive samples, 64 of 71 *P*. *vivax* positive samples and all 4 mixed samples correctly, giving a sensitivity of 93.6% at the species level (Table 3).

**Table 3:** Clinical sensitivity of Truenat Malaria compared to an RDT using expert microscopy as the gold standard. 95% Confidence Intervals (CI) in brackets.

	Truenat Malaria	RDT
**Agreement with expert microscopy positive samples (*P*. *falciparum* positive, *P*. *vivax* positive and mixed infection samples as characterized by expert microscopy)**	**139 (of 140)**	**131 (of 140)**
**Sensitivity (%) using expert microscopy as gold standard**	**99.3 [95.5–99.9]**	**93.6 [87.8–96.8]**

Truenat Malaria detected 32 samples or 11.4% samples as mixed infections (both *P*. *falciparum* and *P*. *vivax* positive). Only 4 of 32 were picked as mixed infection by expert microscopy and 5 were picked as mixed infection by RDT. Of the 32 samples, 19 were detected as *P*. *falciparum* positive only by both expert microscopy and RDT and 4 as *P*. *vivax* positive only by both expert microscopy and RDT. In 19 samples, the *P*. *vivax* parasitic load detected by Truenat Malaria was greater than 3000 genome equivalents/μl. Among the 4 samples, *P*. *falciparum* parasitic load was less than 8000 genome equivalents/μl in all, and as low as 460 genome equivalents/μl in one of the samples. All the raw data for the 281 samples has been provided as Supplementary file (S1 Table).

## Discussion

Malaria is a serious health problem worldwide. The routinely used tests are either time consuming or sometimes show poor sensitivity and specificity. The recent emergence of real-time PCR in infectious disease diagnosis has solved all these concerns by allowing rapid amplification and quantification of target nucleic acid through the use of specific dual labeled fluorescent probes. Since the typical commercial real-time systems cannot be used for point of care applications because of their large size and cost, in the current study we have shown the detection of malaria infections using Truelab Uno^™^, a low cost, portable, point of care device where the real-time PCR occurs on ready to use microchips. As per our estimate, the cost of the test is about one-fifth the cost of a commercial PCR test. Samples are processed one at a time, each sample taking about 1 hour, including sample preparation. One device can process 16 samples per day. Generally, in the national programme a trained Micorscopist examines 60 to 65 blood smears per day. Here, one Technician can process 80 samples per day in five devices with great accuracy. The operational cost of this device would be at par with the existing method when it is upscaled and used in national program. We have reported a validated real-time PCR assay for malaria, Truenat Malaria, whose performance is equivalent to the WHO nested PCR protocol. The Truenat Malaria assay can differentiate between *P*. *falciparum* and *P*. *vivax* in real-time and has a limit of detection between 2 and 5 parasites/μl. This corresponds to a much higher analytical sensitivity than that of the current gold standard microscopy.

A comparison between Truelab Uno^™^ microPCR and commercial thermal cycler showed that the Truelab Uno^™^ microPCR results matched the results obtained using the commercial device. Comparison of the performance of Truelab Uno^™^ microPCR with that of expert microscopy and RDT using a triple-blinded sample panel consisting of 281 clinical samples showed that Truelab Uno^™^ microPCR successfully detected almost all the positive samples. It also detected malarial parasites in 23 samples which were negative by expert microscopy. A recent meta-analysis shows that on average, microscopy detects only half of the malaria infections diagnosed by molecular techniques [20]. In the current study, the microscopic examination was performed by experienced microscopists in a reference laboratory which routinely participates in internal and external quality assurance programs, including international projects on antimalarial drug trials. The same quality of microscopy cannot be assured in peripheral microscopy centers [16]. In actual practice, the lower limit of detection of microscopic examination has been approximated to be around 100 parasites/μl [21], similar to that of RDTs. This may be attributable to operational constraints or technical factors, such as loss of parasites during the staining procedure [22].

The current PCR assay and the RDT test could detect six post-treatment follow-up cases. These samples were not detected by expert microscopy, as the parasites would have got destroyed by the malarial drugs used for treating the patient. While the PCR and the RDT tests could detect these follow-up cases even after the parasite destruction, because the nucleic acids and the protein antigens of the malaria parasites will still be present in the blood circulation, and hence PCR and RDT can detect them.

The Truelab Uno^™^ microPCR detected an additional 27 cases of mixed infections which were reported as either *P*. *falciparum or P*. *vivax* infections by microscopy and RDT. In most of the mixed infection cases, the Ct obtained with the Truenat Malaria assay for the one of the species was higher than the other, translating to one of the species being present in smaller numbers compared to the other in the sample. It has been observed in many previous studies that PCR detects more mixed infections than microscopy [23, 24, 25], especially when one species is numerically dominated by another in a mixed infection as was seen in this study. In 4/32 of the mixed infections detected by PCR from our samples, *P*. *falciparum* was over-looked by both microscopic examination and the RDT. A diagnosis in which *P*. *falciparum* is missed is potentially of grave consequence to the patient. On the other hand, healthcare workers may overcompensate for the shortcomings of microscopy and RDT, leading to presumptive treatment and over-use of anti-malarial drugs and drug resistance [26].

Another problem with the limited sensitivity and unreliable quality of microscopy and RDTs is the impact on control of spread of malaria. Numerous studies have confirmed that sub-microscopic infections are rampant in malaria endemic areas, in cases of both *P*. *falciparum* and *P*. *vivax* infections [25, 27]. Use of microscopy and/or RDTs in epidemiological surveys results in underestimation of the prevalence of low-density infections where the parasitic load is less than 100 parasites/μl [28].

Reservoirs of sub-microscopic, asymptomatic infections are significant hurdles in malaria control programs. Asymptomatic people do not seek treatment and their parasitaemia could not be detected either by microscopy or RDTs even if they were randomly screened. These sub-microscopic pockets of parasites have been shown to be efficient gametocyte producers [29–32] and thus constitute a source of ongoing transmission, potentially accounting for about 20–50% of all human to mosquito transmission [10].

Thus, the goal of current malaria control and elimination strategies is a shift in focus from early diagnosis and treatment of people presenting with malaria-like symptoms, to active surveillance for and treatment of every case, including those who are asymptomatic [33,34]. This necessitates the use of more sensitive techniques for detection of malaria. For measuring progress in reducing malaria transmission, molecular techniques seem to be the best tool for the estimation of parasite prevalence in the general population.

In this study, we have demonstrated a portable and battery-operated molecular diagnostic device and a validated assay for detection of malaria parasites at the species level. With a sub-microscopic limit of detection, this platform could be used for confirmatory malaria diagnosis in peripheral health facilities and also other field testing centres.

**Fig 2:**
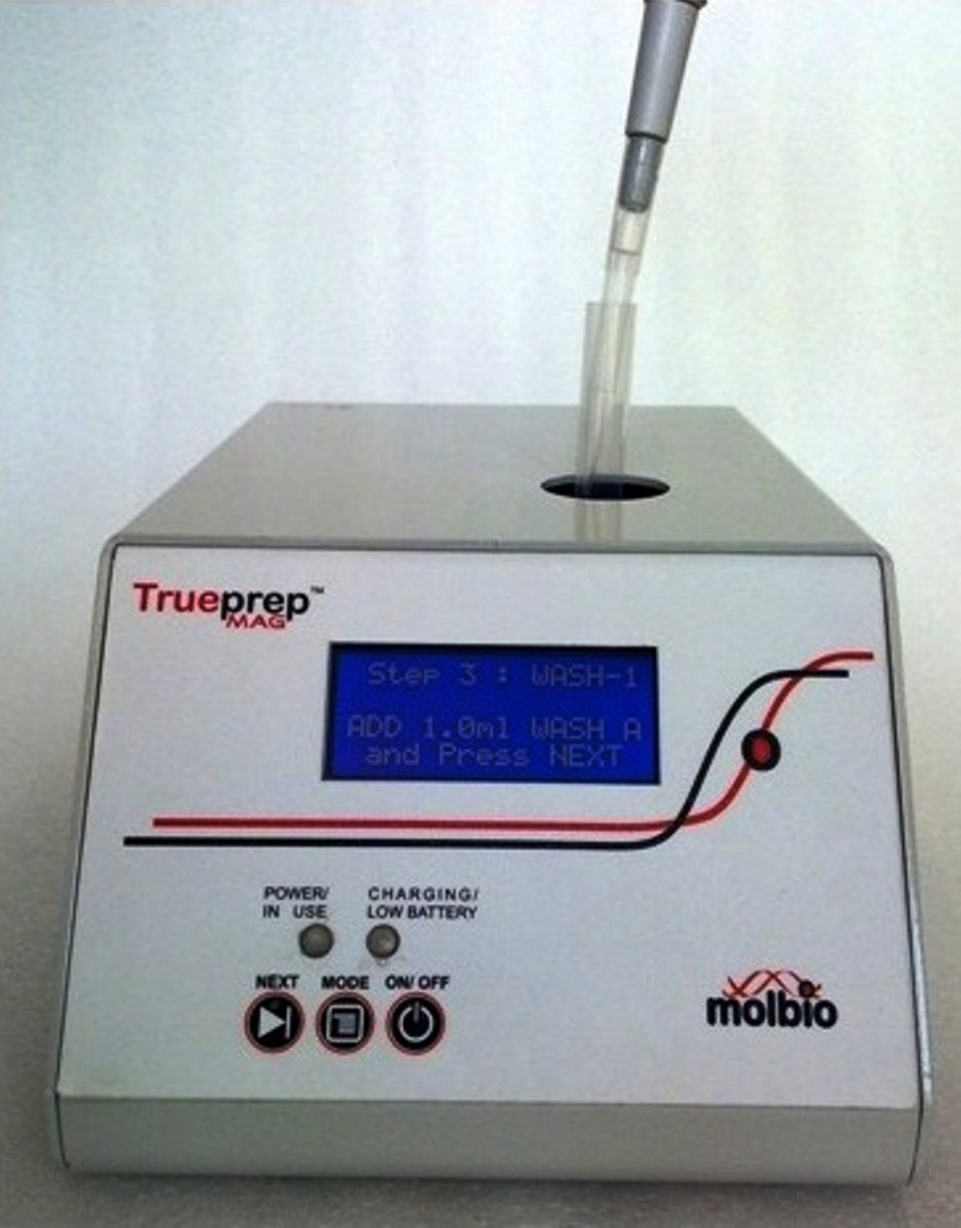
Trueprep MAG^®^ sample processing device, with user adding reagents to the test tube containing the biological specimen.

As this study progressed, bigtec developed a portable and battery-operated sample processing device called Trueprep MAG^®^. The device is a stand-alone nucleic acid extraction unit that does not require the use of any additional equipment like vortexers and heat blocks. It weighs 1.6 kgs and its dimensions are 210 mm x 155 mm x 109 mm (length x breadth x height). The sample processing is done semi-automatically with the user following pipetting instructions as they appear sequentially on the device’s alpha-numeric LCD screen (Fig 2). The chemistry used is a magnetic nano-particle based nucleic acid extraction technique which has been developed in-house, along with the other reagents and buffers required for the sample processing. All reagents are room-temperature stable, same as the Truenat microchips^®^. The device and reagent kits can process a wide-variety of samples like sputum, blood, plasma and serum. The use of the Trueprep MAG^®^ for PCR-based testing in clinical evaluations has been described previously [12, 13] for the extraction of DNA from the sputum of patients suspected of having Tuberculosis. Its performance was established to be comparable to commercially available nucleic acid extraction kits. The use of the Trueprep MAG^®^ in conjunction with the Truelab Uno^®^ and the room-temperature stable reagents developed by bigtec, ensures that “sample-to-result” molecular testing can now be done in point of care settings for diagnosis of infectious diseases like tuberculosis, malaria and other infectious diseases.

For malaria control in particular, an accurate and reliable diagnosis, along with specific information on the species responsible, is useful for both clinicians and epidemiologists in malaria surveillance and elimination programs.

### Limitations of this study

1. Because of the lack of a common reference standard used for the 100 sample panel and the 281 sample panel, the data from both panels could not be combined and performance estimates were calculated separately for both panels. Ideally, the same tests should have been conducted on all samples involved in this study.
2. For the clinical sample panel of 281 samples tested using Truenat^®^ Malaria, expert microscopy and a RDT, a reference nucleic acid test (WHO nested PCR) was not performed. This was because there was not enough blood remaining after performing the microscopic examination and RDT to extract enough DNA to run the Truenat^®^ Malaria test on a commercial thermal cycler and Truelab Uno^®^ and then also performing the nested PCR test. True negative status of the 281 samples could not be established in the absence of nested PCR results. Since expert microscopy failed to detect numerous samples that were positive by Truenat Malaria owing to its higher limit of detection, specificity of the Truenat^®^ Malaria could not be determined using expert microscopy results as a reference for the 281 sample panel.
3. As the development and validation of the Trueprep MAG^®^ platform was not complete when this study was initiated, all sample processing were done using commercially available kits. Multicentric studies need to be initiated to test the performance of the Trueprep MAG^®^ device in the diagnosis of malaria in different field settings.

## Conclusions

The Truenat malaria test on Truelab Uno^®^ microPCR platform was found to be highly useful in rapid, sensitive diagnosis of malaria infections. It was also found to be efficient in detecting cases of mixed malarial infections. Its use could be extended to detection of malaria in active surveillance programs.

## Supporting Information

S1 Table Results of microscopy, RDT and Truenat Malaria testing on 281 sample panel. (XLS)

## Abbreviations

DNA: Deoxyribonucleic acid
PCR: Polymerase Chain Reaction
RDT: Rapid Diagnostic Test
WHO: World Health Organization
NIMR: National Institute of Malaria Research
ICMR: Indian Council of Medical Research
